# Complete Genome Sequence of the Salmonella enterica Serovar Paratyphi A Bacteriophage LSPA1 Isolated in China

**DOI:** 10.1128/genomeA.01011-14

**Published:** 2015-01-15

**Authors:** Weikun Zeng, Pujia Mao, Yu Hong, Mengdie Feng, Zeyang Xu, Fen Huang, Shenrong Jing

**Affiliations:** a Medical Faculty, Kunming University, Kunming, Yunnan, China; b Medical Faculty, Kunming University of Science and Technology, Kunming, Yunnan, China; c Department of Nuclear Medicine, Kunming General Hospital of Chengdu Military Command, Kunming, Yunnan, China

## Abstract

The bacteriophage LSPA1 was isolated from hospital sewage (Kunming, China), and lytic activity was demonstrated against the Salmonella enterica serovar Paratyphi A CMCC50973 strain. This bacteriophage has a 41,880-bp double-stranded DNA (dsDNA) genome encoding 58 coding sequences (CDSs) and belongs to the family Siphoviridae.

## GENOME ANNOUNCEMENT

Paratyphoid fever outbreaks sharply increased in recent years in Asia (1, 2), and its antibiotic resistance is also increasing (3, 4). Understanding Salmonella enterica serovar Paratyphi A phages is important for extending our knowledge and use of phage therapy. The strictly lytic phage LSPA1 was isolated from hospital sewage in Kunming, Southwest China, using the violent strain CMCC50973 of Salmonella Paratyphi A as the host. According to the morphological analysis by transmission electron microscopy, LSPA1 was classified under the family Siphoviridae. DNA sequencing was performed using the Sanger method, with 8× coverage, at the Beijing Genomics Institute (BGI) (Shenzhen, China) using an ABI3700xl automatic sequencer. The sequences were assembled using DNAStar Lasergene version 7.1 (5). LSPA1 contains 41,880 bp and has a G+C content of 49.7%. The potential coding sequences (CDSs) were initially annotated using Glimmer (6) and GeneMark (7). The predicted proteins were analyzed using BLASTp (8). The genome contains 58 predicted genes, with an average gene length of about 653 bp. Thirty-eight of the genes are rightward oriented, while the others are leftward oriented. Fifty-three coding sequences begin with the start codon AUG, while three begin with the start codon GUG and two with UUG.

Based on the predictions, this phage genome contains genes for phage replication, structure, and lysis. Open reading frames (ORFs) were found for putative homing endonuclease, helicase, and DNA polymerase (locus tags LSPA1_03, LSPA1_34, and LSPA1_40, respectively). The ORFs for terminase, head morphogenesis protein, putative tail protein, and tail fiber protein were found (locus tags LSPA1_03, LSPA1_05, and LSPA1_06, LSPA1_31, respectively). No lysogenization, such as site-specific integrases and repressors, was identified. The ORFs for holin and endolysin were found (locus tags LSPA1_52 and LSPA1_53, respectively).

Five phages closely related to LSPA1 are Salmonella phages FSL SP-101 (GenBank accession no. KC139511), wksl3 (GenBank accession no. JX202565), SETP13 (GenBank accession no. KF562864), vB_SenS-Ent3 (GenBank accession no. HG934470), and vB_SenS-Ent2 (GenBank accession no. HG934469).

### Nucleotide sequence accession number.

The complete genome of the Salmonella Paratyphi A bacteriopage LSPA1 has been deposited in GenBank under the accession no. KM272358.
